# Endogenous and fluorescent sterols reveal the molecular basis for ligand selectivity of human sterol transporters

**DOI:** 10.1016/j.jlr.2024.100738

**Published:** 2024-12-31

**Authors:** Laura Depta, Hogan P. Bryce-Rogers, Nienke J. Dekker, Anna Wiehl Bønke, Nicolò Camporese, Mingxing Qian, Yuanjian Xu, Douglas F. Covey, Luca Laraia

**Affiliations:** 1 Department of Chemistry, Technical University of Denmark, Kemitorvet 207, Kgs. Lyngby, Denmark; 2 Department of Developmental Biology, Washington University in St. Louis, School of Medicine, St. Louis, Missouri, USA; 3 Taylor Family Institute for Innovative Psychiatric Research, Washington University in St. Louis, School of Medicine, St. Louis, Missouri, USA

**Keywords:** sterol transport proteins, ligand selectivity, small molecules, natural products, structure activity relationship

## Abstract

Sterol transport proteins (STPs) play a pivotal role in cholesterol homeostasis and therefore are essential for healthy human physiology. Despite recent advances in dissecting functions of STPs in the human cell, there is still a significant knowledge gap regarding their specific biological functions and a lack of suitable selective probes for their study. Here, we profile fluorescent steroid-based probes across ten STPs, uncovering substantial differences in their selectivity, aiding the retrospective and prospective interpretation of biological results generated with those probes. These results guided the establishment of an STP screening panel combining diverse biophysical assays, enabling the evaluation of 42 steroid-based natural products and derivatives. Combining this with a thorough structural analysis revealed the molecular basis for STP-specific selectivity profiles, leading to the uncovering of several new potent and selective Aster-B inhibitors and supporting the role of this protein in steroidogenesis.

The regulation of intracellular cholesterol homeostasis is essential for a healthy human physiology. The functions of this important lipid are diverse, from controlling membrane structure and fluidity to serving as precursors to steroid hormones (1). Intracellular sterol transport proteins (STPs) are responsible for the nonvesicular transport of cholesterol and other sterols between specific organelles, which are divided into three protein families: the ORPs, the STARDs, and the Asters (2, 3, 4).

The oxysterol-binding protein (OSBP) and OSBP-related proteins (ORPs) are key mediators and regulators of lipid transport between the endoplasmic reticulum (ER) and other organelles (5, 6). They share a conserved OSBP-related domain, which has been shown to bind and transfer sterols and other lipids including phosphatidylcholine, ceramide, and phosphatidylinositol phosphates (PIPs) (7). The mammalian steroidogenic acute regulatory protein-related lipid transfer (START) domain (STARD) family contains 15 members. All members contain the StART lipid transfer domain, while only the membrane-targeted STARD1/D3 subfamily and the soluble STARD4 subfamily (STARD4/D5/D6) are reported to bind sterols (8). While STARD1 regulates the delivery of cholesterol from the outer mitochondrial membrane to the inner mitochondrial membrane in steroidogenesis, STARD3 transfers cholesterol from the ER to the late endosomes and mediates interactions between those two organelles (9). The Asters transport cholesterol from the plasma membrane to the ER; however, different expression patterns suggest specific functions (10, 11). Aster-A is highly expressed in the brain, Aster-B in adrenal tissues, and Aster-C in the testis and liver. Aster-A and -C both play a role in autophagosome biogenesis, while Aster-B has been shown to regulate mitochondrial sterol transport and Aster-C has been shown to regulate mTOR activity (12, 13, 14).

So far, information about the specific functions and transport mechanisms within the STP families is mainly based on knock-down and knock-out studies, which sometimes show contradictory results due to their documented functional redundancy (15). Here, an investigation with chemical tools would help to further elucidate the complex interplay of this network. Such a strategy necessitates bioactive ligands with a defined selectivity profile for each protein (16). A growing set of fluorescent sterol derivatives is now commercially available, which are often used interchangeably in the field. However, almost none of these probes have been profiled with regards to their selectivity towards sterol-binding proteins. This can lead to erroneous or incomplete interpretation of biological results generated with these probes. Rectifying this would also enable a more targeted and specific use of these probes to assess the biology of individual or groups of sterol-binding proteins. Recently we reported the initial stages of development of a sterol transport protein screening panel as a tool for the identification and characterization of potent and selective STP inhibitors. The screening platform, as such, enabled the identification of potent and selective OSBP binders, inhibiting retrograde trafficking and reducing Shiga toxin toxicity (17).

Here we describe the profiling of fluorescent sterol-based probes as well as a set of steroid-based natural products, utilizing a more comprehensive STP screening panel, providing the molecular basis for ligand recognition of ten STPs for the first time, combined in one study. The identification of suitable fluorescent probes for the differential STPs enabled the establishment of fluorescence-, Förster (fluorescence) resonance energy transfer (FRET)-, and differential scanning fluorimetry-based assays, facilitating the direct comparison of STP-specific selectivity profiles and evaluation of selective STP inhibitors in a high-throughput manner. We then assess the capabilities of each assay to investigate the binding of cholesterol (bearing in mind the inherent challenges of working with the renowned solubility issues of cholesterol) with each STP in the panel. Next, we describe the screening of 41 sterol- and steroid-based natural products revealing Aster-A and Aster-B as targets for endogenous and synthetic steroid hormones and their precursors as well as STARD5 as the primary target for bile acid derivatives in an aqueous environment. Finally, these results were combined with a detailed structural analysis revealing key residues, which could serve as selectivity handles for the development of highly potent and selective STP inhibitors in the future.

## Materials and Methods

### Chemicals

Lipids were commercially available or synthesized in house (see Supporting information for synthetic procedures). Information regarding supplier, catalog number, and CAS number are available in Supplemental Table S1. Nuclear magnetic resonance spectra were analyzed with MestreNova, v.x64.

### Protein expression constructs

Human ASTER domains of Aster-A(359–547), -B(364–552), and -C(318–504) were subcloned into a pGEX-6p-2rbs vector, thus introducing the cloning artifact ‘GPLGS’ (15) The pGEX-6P-1-GST-OSBP(377–807), pET24b(+)-ORP1(534–950), and pET24b(+)-ORP2(49–480) plasmids were purchased from Genscript. The pET22b_His6_STARD1(66–284) and pET22b_His6_STARD3(216–444) plasmids were a gift from James H. Hurley (University of California) (9). The pHIS_2His6_Thrombin_STARD4(2–205;C75S) (18) plasmid was a gift from Young Jun Im (Chonnam National University). STARD5A was a gift from Nicola Burgess-Brown (Addgene plasmid #42392; http://n2t.net/addgene:42,392;RRID:Addgene_42392).

### Protein expression and purification

The ASTER domains of human Aster-A(359–547), -B(364–552), and -C(318–504) in pGEX-6p-2rps vectors including an N-terminal PreScission-cleavable GST-tag were expressed in *Escherichia coli* (*E. coli*) OverExpress C41 in Terrific Broth medium for 16 h at 18°C after the induction with 0.1 mM IPTG. Cells were harvested at 3,500 *g* for 15 min and lysed by sonication in buffer containing 50 mM Hepes pH 7.5, 300 mM NaCl, 10% (v/v) glycerol, 5 mM DTT, 0.1% (v/v) Triton X-100, and protease inhibitor mix HP plus (Serva). The lysate was purified by affinity chromatography on a GSTrap FF column (Cytiva) using an ÄKTA Start (Cytiva) in buffer containing 50 mM Hepes pH 7.5, 300 mM NaCl, 10% (v/v) glycerol, 5 mM DTT, and 0.01% (v/v) Triton X-100. The GST-tag was cleaved overnight on the column at 4 °C. The Aster sterol-binding domains were further purified by size-exclusion chromatography (SEC) on a HiLoad 16/600 Superdex 75 pg (Cytiva) in buffer containing 20 mM Hepes pH 7.5, 300 mM NaCl, 10% (v/v) glycerol, and 2 mM DTT.

The START domains of human STARD1(66–284), STARD3(216–444), STARD4(2–205; C75S), and STARD5(6–213) harboring an N-terminal His_6_-Tag were expressed in *E. coli* BL21(DE3) in Luria-Bertani Broth medium for approximately 16 h at 18 °C after induction with 0.15 mM IPTG. Cells were harvested at 3,500g for 15 min and lysed by sonication in buffer containing 50 mM Hepes pH 7.5, 150 mM NaCl, 5% (v/v) glycerol, 5 mM DTT, 0.1% (v/v) Triton X-100, and EDTA-free protease inhibitor cocktail (Sigma-Aldrich). The cleared lysate was purified by affinity chromatography on a Ni-NTA Superflow Cartridge (Qiagen) using an ÄKTA Start (Cytiva) in a buffer containing 50 mM Hepes pH 7.5, 150 mM NaCl, 5% (v/v) glycerol, 5 mM DTT. START domains were eluted by using elution buffer containing 50 mM Hepes pH 7.5, 150 mM NaCl, 5% (v/v) glycerol, 5 mM DTT, and 500 mM imidazole. Proteins were further purified by SEC on a HiLoad 16/600 Superdex 75 pg (Cytiva) in buffer containing 20 mM Hepes pH 7.5, 150 mM NaCl, 5% (v/v) glycerol, and 2 mM DTT.

The ORP domain of human OSBP(377–807) in the pGEX-6p-1 vector with an N-terminal PreScission-cleavable GST-tag was expressed in *E. coli* OverExpress C41 in Luria-Bertani Broth medium for 16 h at 18 °C after induction with 0.1 mM IPTG. Cells were harvested at 3,500g for 15 min and lysed by sonication in buffer containing 20 mM Hepes pH 7.5, 300 mM NaCl, 10% (v/v) glycerol, 5 mM DTT, 0.1% (v/v) Triton X-100, and EDTA-free protease inhibitor cocktail (Sigma-Aldrich). The cleared lysate was purified by affinity chromatography on a GSTrap HF column (Cytiva) using an ÄKTA Start (Cytiva) in a buffer containing 20 mM Hepes pH 7.5, 300 mM NaCl, 10% (v/v) glycerol, and 5 mM DTT. OSBP(377–807) was eluted by using elution buffer containing 20 mM Hepes pH 7.5, 300 mM NaCl, 10% (v/v) glycerol, 5 mM DTT, and 10 mM reduced glutathione. Proteins were further purified by SEC on a HiLoad 16/600 Superdex 75 pg (Cytiva) using an ÄKTA Explorer (Cytiva) in a buffer containing 20 mM Hepes pH 7.5, 150 mM NaCl, 10% (v/v) glycerol, and 2 mM DTT.

The ORP domains of human ORP1(534–950) in pET24b(+) and ORP2(49–480) in the pET24b(+) vector including an N-terminal His6-Tag were expressed in *E. coli* BL21(DE3) in terrific broth medium for approximately 16 h at 18 °C after induction with 0.1 mM IPTG. Cells were collected at 3,500 g for 15 min and lysed by sonification in a buffer containing 10 mM Tris-HCl pH 8, 300 mM NaCl, 5% (v/v) glycerol, 2 mM DTT, 0.1% (v/v) Triton X-100, and EDTA-free protease inhibitor cocktail (Sigma-Aldrich). The cleared lysate was purified by affinity chromatography on a Ni-NTA Superflow Cartridge (Qiagen) using an ÄKTA Start (Cytiva) in a buffer containing 10 mM Tris–HCl, pH 8, 300 mM NaCl, 5% (v/v) glycerol, and 2 mM DTT. ORP domains were eluted using elution buffer containing 10 mM Tris-HCl, pH 8, 300 mM NaCl, 5% (v/v) glycerol, 500 mM imidazole, and 2 mM DTT. The proteins were further purified by SEC on a HiLoad 16/600 Superdex 75 pg (Cytiva) using an ÄKTA Explorer (Cytiva) in a buffer containing 10 mM Tris-HCl, pH 8, 150 mM NaCl, 5% (v/v) glycerol, and 2 mM DTT.

### UV-vis absorbance and emission spectroscopy

Measurements were performed on a Tecan Spark Cyto spectrophotometer in an integrated JGS2 quartz 96-well plate from MicQuartz. The measurements were carried out in three different solvents: DCM, methanol, and buffer (20 mM Hepes pH 7.5, 300 mM NaCl, 2 mM DTT), which were used at a final concentration of 40–60 μM, in 200 μl samples. Absorption and emission spectra were corrected to solvent blanks. Absorption measurements are corrected for pathlength, and emission spectra are then normalized to account for variations in gain. All measurements were performed in duplicate and at 25°C.

### Fluorescence polarization

Fluorescence emission measurements as well as fluorescence intensity (FI) and polarization experiments were performed in a buffer composed of 20 mM Hepes pH 7.5, 300 mM NaCl, 0.01% (v/v) Tween-20, 0.5% (v/v) glycerol, and 2 mM DTT in a final volume of 30 μl in black, flat-bottom, nonbinding 384-well plates (Corning). Excitation and emission for each sterol fluorophore are available in Supplemental Table S2.

Fluorescence polarization experiments were performed in a buffer containing 20 mM Hepes pH 7.5, 300 mM NaCl, 0.01% (v/v) Tween-20, 0.5% glycerol, and 2 mM DTT in a final volume of 30 μl in black, flat-bottom, nonbinding 384-well plates (Corning). For k_d_ measurements, fluorophore was incubated with desired concentrations of protein. For competition experiments, 20 nM 22-NBD-cholesterol or 80 nM 25-NBD-cholesterol was mixed with protein and incubated with desired concentrations of screening compounds. The fluorescence polarization signal was measured using a Spark Cyto multimode microplate reader (Tecan) with filters set at 485 ± 20 nm for excitation and at 535 ± 20 nm for emission. The data was analyzed using GraphPad Prism 5. Measured mP values were normalized setting 0% inhibition as the FP signal from the protein + fluorophore control well and 100% as the FP signal from the fluorophore control well. Curves were fitted to the normalized data via nonlinear regression to allow the determination of IC_50_ values. The assay conditions for each protein are available in Supplemental Table S3.

### FI assay

FI experiments were performed in a buffer containing 20 mM Hepes pH 7.5, 300 mM NaCl, 0.01% (v/v) Tween-20, and 2 mM DTT in a final volume of 30 μl in black, flat-bottom, nonbinding 384-well plates (Corning). For k_d_ titrations of protein against fluorophore: fluorophore concentration was kept constant at either 100 nM or 200 nM. The protein was then diluted in a 3-fold fashion from 15 μM or 5 μM. Fifteen microliters of both the fluorophore solution and the protein solution were added to each well and then allowed to incubate for 20 min after centrifuging. A protein-only control titration is made as a control that will be normalized against. The FI signal was measured using a Spark Cyto multimode microplate reader (Tecan) with monochromator set at 280 ± 5 nm and 324 ± 5 nm for excitation and at 450 ± 15 nm for emission. The data was analyzed using GraphPad Prism 5. Measured FI values were normalized against the protein-only control titration. Curves were fitted to the normalized data via nonlinear regression to allow the determination of k_d_ values. In the competitive setup for STARD4, competitor ligands were transferred to the Corning 384-well plate using the LabCyte Echo 550 Liquid Handler. In a single concentration screen, the final ligand concentration was 10 μM and in dose response, a 2-fold dilution across 8 points was used starting with 20 or 10 μM. The final concentration of fluorophore and protein was 80 nM and 120 nM, respectively, with the 27-hydroxycholestatrienol (27-HCTL) and STARD4 stocks being pre-incubated on ice, before adding 30 μl to the plate containing competitor ligands. The plate was then centrifuged and incubated for 20 min, at room temperature, before reading. The FI signal was measured using a Spark Cyto multimode microplate reader (Tecan) with monochromator set at 280 ± 5 nm and 324 ± 5 nm for excitation and at 450 ± 15 nm for emission. The data was analyzed using GraphPad Prism 5. Measured FI values were normalized setting 100% inhibition as the FI signal from the protein-only control well and 0% as the FI signal from the protein + fluorophore control well. Curves were fitted to the normalized data via nonlinear regression to allow the determination of IC_50_ values.

### Differential scanning fluorimetry

Differential scanning fluorimetry (DSF) experiments were performed in a buffer composed of 20 mM Hepes pH 7.5, 300 mM NaCl, and 2 mM DTT in Milli-Q water. Stock solutions of STARD3 and STARD5 were made at a concentration of 5 μM, and STARD4 at 2.5 μM in the Hepes buffer. A LabCyte Echo 550 acoustic dispenser was used to transfer the required amount of DMSO-dissolved ligand into the 384-well plate (LightCycler® 480 Multiwell Plate 384, white). Final concentrations of ligands in a single concentration high-throughput screening are 12.5 μM. For dose response, a 2-fold dilution over 8 points was made starting at concentrations of either 100 μM (STARD3 and 5) or 50 μM (STARD4). This was lowered for compounds clearly showing solubility issues and became compound-specific. After compound addition, 10 μl of protein solutions was manually pipetted to each well using an electronic 12-channel pipette. The plate was then briefly centrifuged before subsequently adding 20 nl of 5000x SYPRO orange (Sigma-Aldrich), with the Echo acoustic dispenser, for a final concentration of 10x SYPRO orange. The FI was measured in a Roche LightCycler 480 II with an initial incubation at room temperature for 10 min before ramping the temperature from 30°C, by steps of 0.2°C, up to 90°C with incubation for 5 s at each step. Melting temperatures were calculated with the Roche TSA analysis program.

### CD assay

All proteins were buffer exchanged to a PBS buffer (10 mM PBS, 138 mM NaCl, 2.7 mM KCl, pH 7.4 in Milli-Q water). The protein concentrations measured are 2.5 μM. In the cases of ligand-protein measurements, protein concentration was maintained at 2.5 μM in the presence of 5 μM ligand. The instrument used for the analysis is a Jasco J-1500 CD Spectrometer. Samples of 200 μl were measured in Quartz SUPRASIL 1 mm Cuvettes (Hellma Analytics). For the CD Spectra, measurements of CD (mdeg), HT (V), and Absorbance (A.U.) were recorded from 250–190 nm, every 1 nm. All measurements were done in duplicate per sample resulting in an averaged measurement. Cell temperature was maintained at 25°C, with a D.I.T. of 2 s, bandwidth of 1 nm, and scanning speed of 50 nm/min. CD Spectra were not baseline corrected and instead reported alongside the PBS blank using Prism 5 (Supplemental Figs. S2 and S4).

For CD variable temperature measurements, measurements of CD (mdeg), HT (V), LD (dOD), and absorbance (A.U.) were recorded at the minima wavelength for each protein (e.g. 210 nm for ORP1), for a range of temperatures from 30 to 75/85°C. The temperature was increased at a rate of 1°C/min with a D.I.T. of 2 s and bandwidth of 1 nm. The melting temperatures were obtained using the Jasco instrument analysis software while the spectra were replotted using Prism 5 to illustrate the unfolding of the secondary structure (Supplemental Fig. S4).

## Sterol transfer assay

### Preparation of vesicles - TopFluor setup

1,2-dioleoyl-sn-glycero-3-phosphocholine (DOPC, Avanti Polar Lipids, 850375C) was prepared in chloroform (10 mg/ml); 23-(dipyrrometheneboron difluoride)-24-norcholesterol (TopFluor® Cholesterol, Avanti Polar Lipids, 810255) and *N*-(lissamine rhodamine B sulfonyl)-1,2-dihexadecanoyl-sn-glycero-3-phosphoethanolamine (triethylammonium salt) (Rh-DHPE, Invitrogen, L1392) were prepared in methanol (100 μM). The acceptor liposomes (LA) consist of DOPC only while the donor liposomes (LD) consist of a mixture of DOPC:TF-Chol:Rh-DHPE (99:0.5:0.5). The solvent was evaporated under a stream of nitrogen, followed by drying under vacuum overnight. The lipid films were hydrated to a final concentration of 60 μM using buffer containing 20 mM Hepes pH 7.5, 300 mM NaCl, and 2 mM DTT. To fully dissolve the lipid films, the solutions were vortexed and sonicated for 5 min in a 40°C water bath, followed by five freeze and thaw cycles in liquid nitrogen. Extrusion through a polycarbonate membrane (13 times, 0.1 μM pore size, Avanti Polar Lipids) at 40°C yielded homogenous unilamellar vesicles, which were kept on ice and used on the same day.

### Preparation of vesicles - DHE setup

A 2 mM stock solution of dehydroergosterol (DHE) in absolute ethanol was prepared from 1 mg solid (Avanti Polar Lipids, #810253). Dansyl-PE (1 ml, 1 mg/ml) in chloroform was obtained from Avanti Polar Lipids (#810333A). A stock solution of DOPC in chloroform (10 mg/ml) had previously been prepared from a 25 mg/ml solution (Avanti Polar Lipids #850375C). The following steps all took place in glass-vials covered in aluminum foil, to keep the light-sensitive lipids in the dark as much as possible: The stock solutions were mixed in a molar ratio of 90/10 DOPC/DHE or 80/10/10 DOPC/DHE/Cholesterol for the donor vesicles and 97.5/2.5 Dansyl-PE for the acceptor vesicles to a final volume of 1 ml in chloroform. Evaporation of the solvent under a stream of nitrogen, followed by drying under vacuum overnight afforded the dried lipid films. The lipid films were hydrated in a buffer of 20 mM Hepes pH 7.5, 300 mM NaCl, and 2 mM DTT to a final concentration of 260 μM. The solutions of the lipid films were vortexed extensively until full hydration was observed and sonicated for five min in a 40°C water bath followed by five freeze-thaw cycles (−196°C → 40°C). Homogeneous unilamellar vesicles were obtained by extrusion 13 times through a polycarbonate membrane (0.1 μm pore size, Avanti Polar Lipids) at 40°C. Solutions were kept on ice and used on the same day as preparation.

### Microplate-based cholesterol transfer assay

In a nonbinding clear-bottom 96-well plate (Greiner Bio-one, cat# 655906), wells were prepared as follows.

## Topfluor setup

### Preparation for run

A master mix of donor and acceptor liposomes was made in buffer (20 mM Hepes pH 7.5, 300 mM NaCl, 2 mM DTT) affording a final assay concentration for both donor and acceptor as 8 μM (total liposome concentration 16 μM) and a final assay volume of 100 μl. For compound containing runs, protein and compound were incubated at a concentration 20 times the desired assay concentration for 15 min.

### The run

Ninety-five microliters of liposome mixture was then added to each well (maximally 6 wells per run). FI measurements were performed in a Tecan Spark Cyto plate reader at 25 °C, measuring from the bottom at 10 s intervals. The excitation filter was set at 485 ± 25 nm and the emission filter was set to 590 ± 20 nm. After approximately 2 min, the measurement was paused, the plate was ejected, and 5 μl protein (or protein + compound pre-mix) was added as quickly as possible and mixed with a pipette to a desired final concentration. The measurement was continued, and the total measuring time was 12 min. Data was normalized to I_0_ of the donor + acceptor (before adding protein) and plotted in GraphPad Prism 5.

## DHE setup

### Preparation for run

A master mix of donor and acceptor liposomes was made in buffer (20 mM Hepes pH 7.5, 300 mM NaCl, 2 mM DTT) affording a final assay concentration for both donor and acceptor as 12.5 μM (total liposome concentration 25 μM) and a final assay volume of 100 μl. For compound containing runs, protein and compound were incubated at a concentration 20 times the desired assay concentration for 15 min.

### The run

Ninety-five microliters of liposome mixture was then added to each well (maximally 6 wells per run). FI measurements were performed in a Tecan Spark Cyto plate reader at 25 °C, measuring from the bottom at 10 s intervals. The excitation filter was set at 340 ± 20 nm and the emission filter was set to 535 ± 20 nm. After approximately 2 min, the measurement was paused, the plate was ejected, and 5 μl protein (or protein + compound pre-mix) was added as quickly as possible and mixed with a pipette to a desired final concentration. The measurement was continued, and the total measuring time was 12 min. Data was normalized to I_0_ of the donor + acceptor (before adding protein) and plotted in GraphPad Prism 5. The final assay setup and final assay concentrations for each protein are available in Supplemental Table S3.

### Molecular modeling

Software: Maestro version 13.3.121, MMshare Version 5.9.121, Release 2022-3, Platform Windows-x64.

Crystal structure PDB source files were obtained from the Protein Data Bank (PDB IDs in Supplemental Table S5). Protein preparation was carried out using the default workflow with a few minor adjustments. Namely, in the Preprocess workflow, create disulphide bonds, fill in missing loops (using Prime) were selected, and setting the variation of het states (using Epik) to pH range 7.5 ± 0.5. For the H-bond assignments workflow, H-bonds were assigned using PROPKA at pH 7.5. In the final Minimize and Delete waters workflow, a restrained minimization was performed with a convergence of 0.3 Å to heavy atoms. If there existed waters in the binding site, the preparation was optimized by running a validation test on the binding of the cognate ligands. Ligand preparation was carried out on all possible stereoisomeric forms of the ligand, which is desalted and ionized at pH 7.5 ± 0.5 using Epik. Force field used is OPLS4.

For initial screenings of crystal structures with ligands, receptor grid generation was carried out centroid on the co-crystallized ligand. High throughput ligand docking was carried out using Glide, utilizing standard precision, with flexible ligands. The settings allowed for the sampling of nitrogen inversions and ring conformations. Epik state penalties were applied to docking scores. Three poses for each ligand were generated, allowing for more precise accounts of ligand-protein viability. Postdocking minimization and strain correction were also applied to the scoring of each ligand. Pose views were sampled in correlation to the Docking scores. For Induced Fit workflows, the binding domain is centroid on the resident ligand. Ligands are free to sample variations in ring conformation. In glide docking, the protein preparation-constrained refinement is selected for with maximally 20 poses to be generated. Prime refinement is within 5 Å of ligand poses and the Glide redocking is at standard precision. Pose analysis was performed on all examples provided from the simulation. Key considerations were on the retention of any significant position, residue interactions, and orientations that were observed as median averages. Details for the crystal structures used for each protein are available in Supplemental Table S5.

## Results

### Sterol-binding domains show differential binding affinity towards fluorescent sterol-based probes

**Fig. 1:**
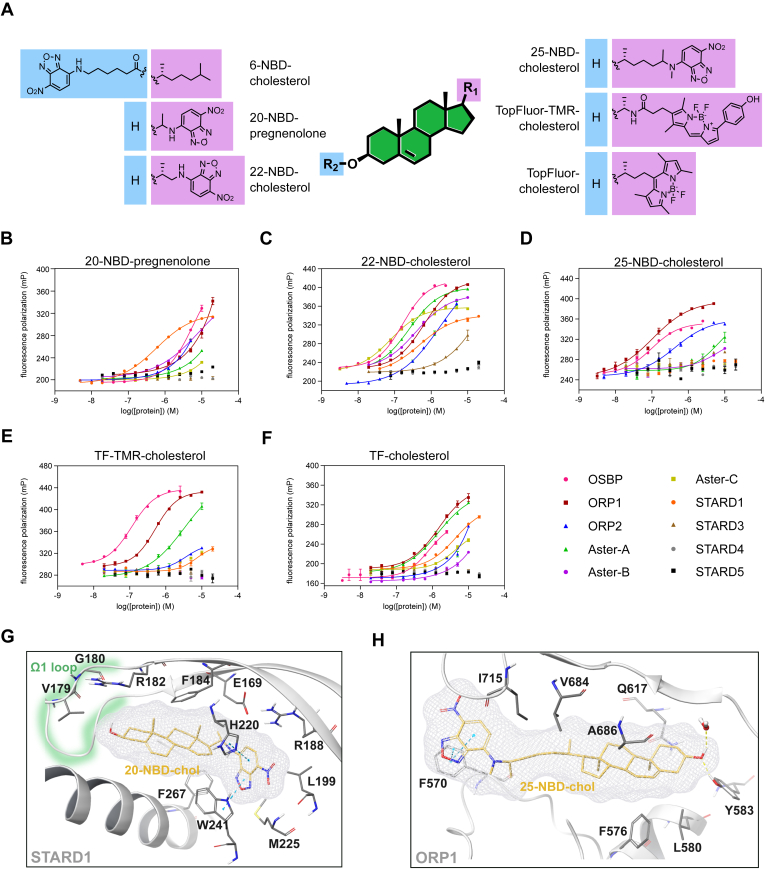
Investigation of sterol-based fluorescent probes towards their binding affinity against sterol transport proteins. (A) Representation of the chemical structures of the sterol-based NBD-labeled fluorescent probes. Direct titration of sterol-based fluorescent probes against increasing protein concentrations enables the determination of k_d_ by monitoring changes in fluorescence polarization for (B) 20-NBD-pregnenolone, (C) 22-NBD-cholesterol, (D) 25-NBD-cholesterol, (E) TopFluor-TMR-cholesterol, and (F) TopFluor-cholesterol. Experimental points were measured in duplicates on each plate and were replicated in n = 3 biologically independent experiments. Error bars indicate s.e.m. (G) Predicted binding pose of 20-NBD-pregnenolone into the crystal structure of STARD1 (pdb: 3p0l) indicating possible interactions with W24 and H220. (H) Predicted binding pose of 25-NBD-chol into the ORP1 crystal structure (pdb: 5zm5) highlighting possible interactions with Y583 and F570.

We initially sought to assess the binding selectivity of different fluorescent sterols against STPs. To do this, we employed our recently developed STP screening panel comprising OSBP, ORP1, and ORP2 as members of the ORP family, STARD1, as well as Aster-A, Aster-B, and Aster-C. To further expand this screening platform, we expressed and purified the sterol-binding domains of STARD3, STARD4, and STARD5 harboring an N-terminal His_6_ tag. CD spectroscopy confirmed the correct folding of all ten STPs (for CD spectra, please see Supplemental Fig. S1). We screened six different sterol-based fluorophores against each sterol-binding domain (Fig. 1A). Their fluorescence properties (excitation and emission spectra, Supplemental Fig. S2) enabled the determination of dissociation constants (k_d_) by direct titration against increasing protein concentrations and monitoring changes in FI or fluorescence polarization (Table 1).

**Table 1:** Summary of binding affinities of sterol-based fluorescent probes against ten different sterol transport proteins

	OSBP	ORP1	ORP2	Aster-A	Aster-B	Aster-C	STARD1	STARD3	STARD4	STARD5
	FP - k_d_ [nM]	FP - k_d_ [nM]	FP - k_d_ [nM]	FP - k_d_ [nM]	FP - k_d_ [nM]	FP - k_d_ [nM]	FP - k_d_ [nM]	FP - k_d_ [nM]	FP - k_d_ [nM]	FP - k_d_ [nM]
6-NBD-chol	>5,000	>5,000	4,560	>5,000	>5,000	>5,000	>5,000	>5,000	>5,000	>5,000
20-NBD-preg	4,042	>5,000	>5,000	>5,000	4,818	>5,000	487	>5,000	>5,000	>5,000
22-NBD-chol	149	322	4,648	357	388	63	573	>5,000	>5,000	>5,000
25-NBD-chol	105	114	588	>5,000	>5,000	>5,000	>5,000	>5,000	>5,000	>5,000
TF-TMR-chol	132	609	2,920	3,262	>5,000	>5,000	>5,000	>5,000	>5,000	>5,000
TF-chol	559	1,162	>5,000	1,472	>5,000	4,805	4,803	>5,000	>5,000	>5,000

FP = fluorescence polarization; na = not applicable. All data is the mean of three biologically independent experiments.

6-NBD-cholesterol showed weak or no binding of the STPs (Supplemental Fig. S3), most likely due to the fact that sterols are predicted to bind “head-first” to all STPs and thus substitution on the A-ring is not tolerated. 20-NBD-pregnenolone (20-NBD-preg) only binds STARD1 tightly, with a >10-fold selectivity over other STPs (Fig. 1B). Furthermore, we observed a STARD1-specific increase in fluorescence upon 20-NBD-preg binding (Supplemental Fig. S4), which was not the case for other NBD-chol probes, suggesting a distinct binding mode (vide infra) (19). 22-NBD-cholesterol (22-NBD-chol) appears to be the most universal probe, showing a k_d_ below 500 nM for six out of ten STPs (Fig. 1C). Most noticeably, it does not show any binding affinity towards STARD3/4/5. On the other hand, 25-NBD-cholesterol (25-NBD-chol), which harbors a longer linker between the cholesterol core and NBD-group, shows selective binding to the ORP family proteins OSBP, ORP1, and ORP2 (Fig. 1D). TF-TMR-cholesterol (TF-TMR-chol) shows preferred binding to OSBP (k_d_ = 132 nM) while having a lower binding affinity towards the other STPs, suggesting a potential utility as a selective fluorescent probe (Fig. 1E). This result is consistent with the recently reported importance of an amide linker between the sterol core and sidechain, which is suggested to interact with Thr491, conferring tight OSBP binding and high selectivity (17). Furthermore, TF-TMR-chol could serve as an alternative probe to the NBD-labeled probes for different purposes, since it is excited and emits at different wavelengths (Supplemental Fig. S2).

In contrast, TF-chol shows an overall weaker binding to the STPs suggesting that a longer linker between the cholesterol core and the BODIPY group might be preferred. However, STARD3, STARD4, and STARD5 do not bind any of the side chain-labeled fluorescent probes, suggesting that they might prefer smaller ligands because of their short and narrow binding pockets. While crystal structures of several STPs have been reported to date, only OSBP, ORP1, as well as murine Aster-A and Aster-C have been crystallized in complex with a ligand binding in the sterol-binding pocket. To obtain insights into the specific binding modes of selected fluorophores and to rationalize their biological effect, we performed docking studies utilizing an induced fit-based approach. Interestingly, the most conserved poses of 20-NBD-preg docked into the crystal structure of STARD1 (pdb: 3p0l (20)) with a head-out orientation, where the NBD-group binds deep into the sterol-binding pocket. The modeling predicts interactions of the NBD-group with H220 and W27 providing a plausible explanation for its turn-on fluorescence upon binding to STARD1 (Fig. 1G). The modeling of 25-NBD-chol into the ORP1 crystal structure (pdb: 5zm5 (21)) suggest a head-in orientation with the hydroxy-group interacting with a water molecule as well as Y583. Additionally, the NBD-group is predicted to interact with F570 located at the opening of the sterol-binding pocket, which is conserved among the ORPs (OSBP F440; ORP2 F69) and thereby explaining their tight binding to 25-NBD-chol (Fig. 1H).

### Thermal shift assays and sterol transfer assays reveal 25-HCTL and 27-HCTL as potent STARD protein inhibitors

**Fig. 2:**
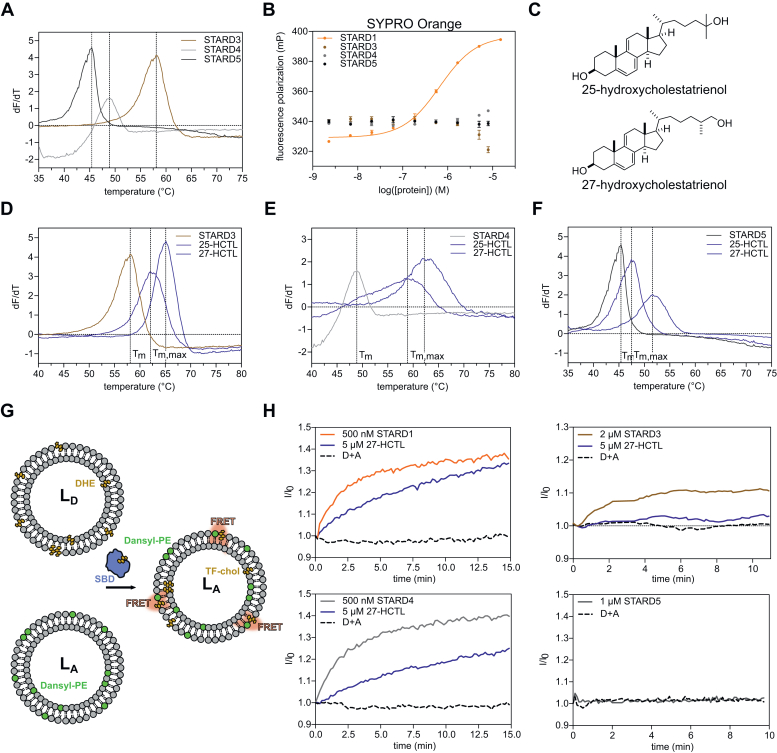
25-HCTL and 27-HCTL stabilize STARD3, STARD4, and STARD5. (A) Thermal stability of STARD3, STARD4, and STARD5 assessed by differential scanning fluorimetry. (B) Differences in fluorescence polarization upon titration of SYPRO Orange against increasing protein concentrations showing binding of STARD1 to SYPRO Orange. (C) Chemical structures of 25-hydroxycholestatrienol (25-HCTL) and 27-hydroxycholestatrienol (27-HCTL). (D) Thermal stabilization of STARD3 incubated with single concentrations of 25-HCTL and 27-HCTL. (E) Thermal stabilization of STARD4 incubated with single concentrations of 25-HCTL and 27-HCTL. (F) Thermal stabilization of STARD5 incubated with single concentrations of 25-HCTL and 27-HCTL. (G) Schematic of the FRET-based sterol transport assay used for the STARDs. (H) Transport of dehydroergosterol (DHE) by STARD1/3/4/5, and its inhibition by 27-HCTL, as assessed by a FRET assay. All data represents a representative experiment from three biological replicates (n = 3). A, acceptor liposome; D, Donor liposome.

Due to the lack of a suitable fluorescent probe for the development of a competitive fluorescence-based assay for STARD3, STARD4, and STARD5, we investigated DSF. In previous studies, this method has already proven to be useful for the screening of potent and selective Aster inhibitors (22, 23). Upon incubation of the STARD proteins with SYPRO Orange, we could observe usable melting curves for STARD3, STARD4, and STARD5 (Fig. 2A). However, for STARD1, no melting curve was observed. Direct titration of SYPRO Orange against increasing concentrations of the individual STARD proteins revealed its binding affinity to STARD1 in the nanomolar range and thereby explaining interference with the DSF assay (Fig. 2B). The same effect was observed for ORP1 and ORP2, however, not for Aster-A-C, thus proving the correlation between SYPRO Orange binding and the usability of DSF for the specific STP (Supplemental Fig. S5). Next, we sought to investigate intrinsically fluorescent sterol probes. As DHE had very poor solubility and would not be suitable as a tracer in fluorescence-based assays where it has not previously been integrated into membranes, we tested single concentrations of the intrinsically fluorescent oxysterols 25-HCTL and 27-HCTL (Fig. 2C) against STARD3-5, as we predicted that their smaller size may be better tolerated by this class of STPs (24, 25). We observed a strong stabilization of all three proteins by 27-HCTL, while 25-HCTL preferentially stabilizes STARD3 and STARD4 (Fig. 2D–F and Table 1). As an orthogonal assay, we employed variable temperature measurements using CD as a readout to confirm the binding of 25-HCTL and 27-HCTL to the specific STPs (Supplemental Fig. S6). We expanded the profiling of 25- and 27-HCTL by screening them as competitive inhibitors in FP assays against the remaining STPs (Table 2). Interestingly, 27-HCTL seems to be more promiscuous towards STPs (Table 2), which could be explained by its slightly longer and more flexible side chain, a result of the position of the hydroxyl group on the terminal methyl in the sterol side chain. This gave early indications that small structural changes can lead to differential selectivity profiles towards the STPs.

**Table 2:** Summary of the binding affinities of 25- and 27-HCTL measured by using a competitive FP assay as well as a thermal shift assay

	OSBP	ORP1	ORP2	Aster-A	Aster-B	Aster-C	STARD1	STARD3	STARD4	STARD5
	FP - IC_50_ [nM]	FP - IC_50_ [nM]	FP - IC_50_ [nM]	FP - IC_50_ [nM]	FP - IC_50_ [nM]	FP - IC_50_ [nM]	FP - IC_50_ [nM]	ΔT_m,max_ [ºC]	ΔT_m,max_ [ºC]	ΔT_m,max_ [ºC]
25-HCTL	238	1,303	3,115	>10,000	6,960	8,255	3,121	5.9	10.3	3.2
27-HCTL	292	777	1,983	625	2,242	3,060	2,019	7.4	13.8	8.6

FP = fluorescence polarization. ΔT_m,max_ refers to the maximal stabilization of a protein by the compound across all concentrations. All data is the mean of three biologically independent experiments.

Next, we employed an in vitro assay based on FRET to evaluate the effect of 27-HCTL on the sterol transfer functions of the STARD proteins. Here, the transfer of the fluorescent cholesterol analog DHE between a donor (L_D_) and an acceptor liposome (L_A_) is followed. The L_D_ liposomes contain DHE, while the L_A_ contain Dansyl-PE. The STP-mediated transfer of DHE from the L_D_ to the L_A_ results in DHE and Dansyl-PE forming a FRET pair and thereby leading to an increase in FI signal (Fig. 2G) (18). Notably, we could observe a transport of DHE by STARD1, STARD3, and STARD4 only, while for STARD5, no increase in fluorescence was observed. Those results indicate that STARD5 might not be involved in cholesterol transport but in the transport of other ligands. We were able to observe a decreased FRET signal for STARD1, STARD3, and STARD4 incubated with 27-HCTL, indicating its inhibition of sterol transport.

### Intrinsic fluorescence of 25-HCTL and 27-HCTL reveals their high affinity binding to the STARDs

**Fig. 3:**
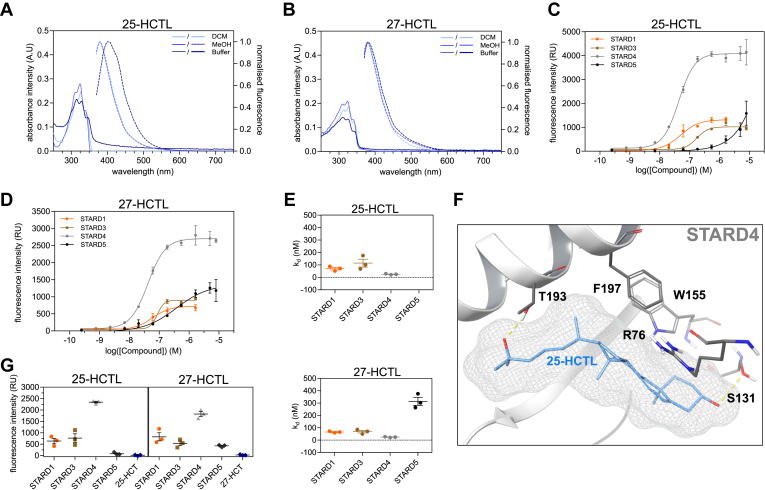
Inherent fluorescence of 25- and 27-HCTL enables the determination of dissociation constants. (A) Excitation (solid lines) and emission (dashed lines, excitation at 325 nM) spectra of 25-HCTL in DCM, methanol, or Hepes buffer. (B) Excitation and emission (excitation at 325 nM) spectra of 27-HCTL in DCM, methanol, or Hepes buffer. (C) Titration of 25-HCTL against STARD1, STARD3, STARD4, and STARD5 assessed by FI using an excitation wavelength of 280 nm and an emission wavelength of 450 nm. (D) Titration of 27-HCTL against STARD1, STARD3, STARD4, and STARD5 assessed by FI using an excitation wavelength of 280 nm and an emission wavelength of 450 nm. (E) Dissociation constants of 25- and 27-HCTL for STARD1, STARD3, STARD4, and STARD5. (F) Representation of the most conserved binding pose of 25-HCTL docked into the crystal structure of STARD4 (pdb: 6l1d). (G) Representation of fluorescence intensity windows for 80 nM 25-HCTL and 27-HCTL incubated with the different STARD proteins.

As no suitable fluorescent probe for STARD3, STARD4, and STARD5 was identified, the intrinsic fluorescence of 25-HCTL and 27-HCTL was investigated. UV-vis and fluorescence spectra revealed excitation peaks around 325 nm and emission peaks around 420 nm. The comparison between spectral data of 25- and 27-HCTL measured in buffer, methanol, and DCM showed sensitivity to the environment resulting in changes in intensity and shifts in emission wavelength (Fig. 3A, B). Following this, we hypothesized that binding into a hydrophobic sterol-binding domain could result in changes in the emission spectra. Upon titration of increasing sterol-binding domain concentrations, we observed an increase in fluorescence for all four STARD proteins (Supplemental Fig. S7A, B). To confirm the specificity of this effect, we employed FRET measurements between the protein’s tryptophan residues and the intrinsically fluorescent sterol, as previously described to confirm specific binding of macarangin B enantiomers to OSBP (26). Using an excitation wavelength of 280 nm and an emission wavelength of 450 nm, we were able to measure the specific k_d_’s of 25-HCTL and 27-HCTL for the STARD proteins (Table 3 and Fig. 3C, D). The results correlate with the previously measured IC_50_ (STARD1) and the ΔT_m,max_ values (STARD3, STARD4, STARD5) showing tight binding of all four STARD proteins to 27-HCTL, while 25-HCTL binds STARD1, STARD3, and STARD4 only (Fig. 3e). Based on these results, we investigated the usability of both ligands as fluorescent probes for the development of competitive binding assays by monitoring changes in FRET-based FI. While 80 nM probe incubated with 120 nM STARD4 gave a sufficient assay window and a Z-factor of 0.8, we could not observe sufficient windows for the other STARD proteins (Fig. 3G and Supplemental Fig. S7C, D) suggesting that 25- and 27-HCTL are suitable fluorescent probes for STARD4 only. The use of higher protein concentrations did not improve the assay window. Modeling of 25-HCTL into the STARD4 crystal structure (pdb: 6l1d) (18) revealed a possible explanation for the much higher increase in FI upon binding to STARD4 in comparison to the other STARD proteins (Fig. 3F). The most conserved poses predict a head-in conformation of 25-HCTL in the sterol-binding pocket and close-proximity of the ligand to W155, promoting direct FRET between ligand and protein.

**Table 3:** Comparison of the binding affinities of cholesterol to the STPs measured by using a competitive FP assay as well as DSF- and CD-based thermal shift assays

	OSBP	ORP1	ORP2	Aster-A	Aster-B	Aster-C	STARD1	STARD3	STARD4	STARD5
FP/FI - IC_50_ [nM]	37	>10,000	>10,000	583	1,288	>10,000	>10,000		>10,000	
DSF - ΔT_m,max_ [ºC]								2.1	1.6	0.1
CD - ΔT_m,max_ [ºC]		2.9	2.5	6.7	8.1	2.3	7.3	2.5	1.1	−0.5

FP = fluorescence polarization. ΔT_m,max_ refers to the maximal stabilization of a protein by the compound across all concentrations. All data is the mean of three biologically independent experiments.

### The cholesterol paradigm: cholesterol shows different thermal stabilizations of STPs in aqueous solution

**Fig. 4:**
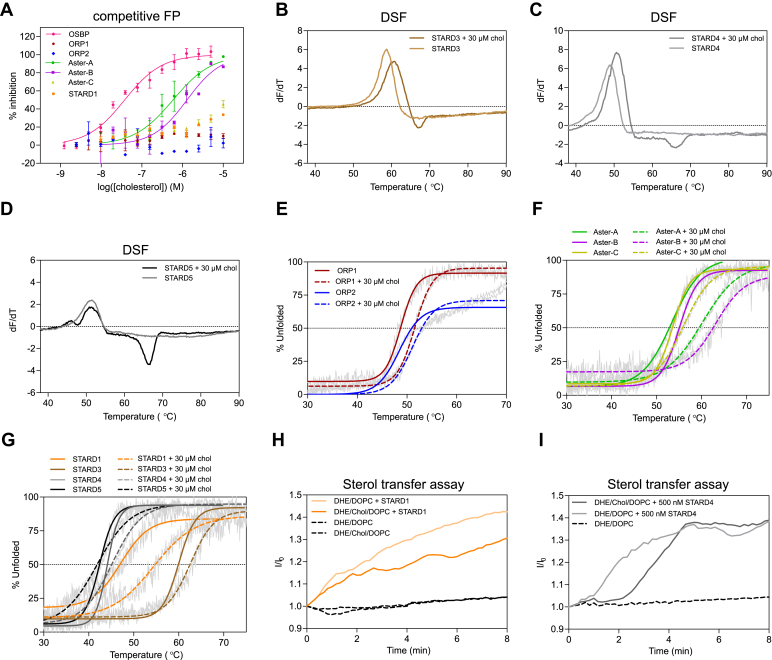
Cholesterol shows different thermal stabilizations of STPs in aqueous solution. (A) Dose-dependent inhibition of NBD-chol binding to OSBP (pink), ORP1 (dark red), ORP2 (blue), Aster-A (green), Aster-B (purple), Aster-C (gold), and STARD1 (orange) by increasing cholesterol concentrations assessed by FP. One representative experiment is shown from three independent experiments (n = 3). Thermal stabilization of STARD3 (B), STARD4 (C), and STARD5 (d) by 30 μM chol assessed by DSF. Thermal stabilization of ORP1/2 (E), AsterA-C (F), and STARD1/3/4/5 (G) by 30 μM cholesterol assessed by CD. Transport of dehydroergosterol (DHE) by STARD1 (H) and STARD4 (I) comparing liposomes with and without embedded cholesterol, as assessed by a FRET assay. All data represents a representative experiment from two biologically independent replicates (n = 2). D, Donor liposome; A, acceptor liposome.

With a maximal solubility of around 4.7 μM and a critical micelle concentration of 25–40 nM in aqueous solution, the determination of accurate binding constants of cholesterol to proteins in solution is challenging. Furthermore, cholesterol micelles are, in addition to hydrophobic repulsion by the solvent, stabilized by strong intermolecular interactions making the extraction of single cholesterol molecules very challenging (27). Aware of these challenges and limitations, we sought to test the capabilities of our biophysical STP screening platform to measure cholesterol binding. Within the last decades, several studies report the binding of cholesterol to STPs. However, to the best of our knowledge, there are no reports comparing the direct binding of cholesterol to all ten proteins in the same study. While NBD-labeled probes were used for the ORPs, the Asters, and STARD1, we used 27-HCTL as a fluorescent probe for STARD4. For STARD3, STARD4, and STARD5, we used DSF as an alternative and orthogonal assay. Furthermore, we utilized a CD-based thermal shift assay as a comparative method across all proteins (Table 3). In the competitive FP assay, we used 3% ethanol to improve cholesterol solubility. Nevertheless, we observed severe solubility issues using concentrations over 10 μM cholesterol. While OSBP (IC_50_ = 37 nM), Aster-A (IC_50_ = 583 nM), and Aster-B (IC_50_ = 1,288 nM) show binding affinities consistent with previous studies (13, 28), we observed weak or no binding for the other STPs to cholesterol (Fig. 4A) under 10 μM. Notably, STARD1 does not tolerate ethanol concentrations higher than 0.3% making the determination of an IC_50_ value with this method for cholesterol impossible. Additionally, the DSF-based thermal shift assay reveals weak stabilization of STARD3 (ΔT_m,max_ = 2.1°C) and STARD4 (ΔT_m,max_ = 1.6 °C) and no stabilization of STARD5 (ΔT_m,max_ = 0.1°C) employing 30 μM cholesterol (Fig. 4B–D). Interestingly, we did not observe solubility issues up to 30 μM cholesterol, suggesting that the increasing temperature during the measurement influences the solubility and critical micelle concentration of cholesterol in aqueous buffer. However, melting curves for each protein showed a distinct negative peak at 68°C, which can be assigned to the denaturation of cholesterol micelles upon comparison with its melting curve without addition of protein (Supplemental Fig. S8A). Next, we employed CD variable temperature measurements to compare the thermal stabilization of all proteins by cholesterol. Consistent with the competitive FP data and the literature reporting weak binding of cholesterol in aqueous solution, ORP1 and ORP2 only show weak stabilization by cholesterol (Fig. 4E) (29, 30). Interestingly, the CD measurements confirm strong binding of Aster-A and Aster-B, while Aster-C shows weaker binding indicating significant differential structural properties (Fig. 4F). Within the STARD family only STARD1 shows a strong stabilization by cholesterol while STARD3/4/5 show weak or no stabilization (Fig. 4G). Furthermore, the CD spectra of STARD1 reveal significant structural changes upon binding of cholesterol (Supplemental Fig. S8B), which could be explained by STARD1’s previously reported molten globule character (31). All in all, our results show different binding affinities of cholesterol to the STPs in aqueous solution. We chose STARD1 and STARD4 to investigate if the observed trends are transferred in a liposome-based environment in the liposome FRET assay. STARD1’s transport of DHE is reduced by nearly 50% when cholesterol is also incorporated in the liposomes, indicating competition of DHE and cholesterol (Fig. 4H). STARD4’s DHE transport seems to be delayed, however still reaching 100% DHE transfer, suggesting a higher binding affinity of DHE to STARD4 in comparison to cholesterol (Fig. 4I).

### Biophysical screening platform reveals differential selectivity profiles of sterol transport proteins

To gain a deeper insight into the individual selectivity profiles of the STPs, we employed the biophysical screening platform to screen a set of 41 natural and synthetic steroidal compounds, which can be further divided into four different classes. We tested 9 oxysterols, 26 steroid hormones and hormone precursors, 5 cholic acid derivatives, as well as the phytosterol β-sitosterol. First, a single concentration (10 μM) screen was performed to identify potential STP binders (Supplementary Dataset 1). We chose 50% inhibition for the competitive assays and a ΔT_m_ of two degrees for the DSF assay as a cut-off for further investigations. Compounds that fulfilled these criteria were tested in dose response against all ten STPs (Table 4). Interestingly, in this compound library, we did not identify STARD4 ligands with affinities <10 μM. However, the results show that a differential hydroxylation pattern on the cholesterol core can result in differential selectivity profiles towards the STPs. OSBP binds most of the oxysterols with high affinity. 4*β*-HC, 7*ɑ*-HC, and 7*β*-HC selectively bind to OSBP showing that hydroxylation’s at the sterol core are more tolerated in OSBP than the other STPs (32). Furthermore, 20(*S*)-HC binds OSBP with an IC_50_ of 41 nM showing a > 20-fold selectivity ratio towards the other STPs. Retrospectively, the high affinity and selectivity of 20(*S*)-HC to OSBP may provide an explanation for the Golgi accumulation of a fluorescent alkynyl derivative of 20(*S*)-HC observed in previous studies (33). In contrast, side chain-modified oxysterols show promiscuous binding to several STPs. However, OSBP does not show binding to 22(*R*)-HC while ORP1, ORP2, as well as Aster-B do, suggesting an unfavorable positioning of the OH-group in the OSBP-binding pocket (34). An overlay of the predicted poses of 22(*R*)-HC and 25-HC in the recently published crystal structure of OSBP (pdb: 7v62) (35) reveals an explanation for their differential binding affinities (Supplemental Fig. S9B). While both ligands are predicted to bind in the “head-in” conformation, 25-HC possibly interacts with T491 as well as D453 and K577. The hydroxylation at position 22 in the (*R*)-configuration seems to result in a different orientation of the sterol core hindering favorable interactions with the key residues in the OSBP-binding pocket.

**Table 4:** Summary of the binding affinities of selected STP hits measured by using a competitive FP/FI assay as well as a thermal shift assay

	OSBP	ORP1	ORP2	Aster-A	Aster-B	Aster-C	STARD1	STARD3	STARD5
	FP - IC_50_ [nM]	FP - IC_50_ [nM]	FP - IC_50_ [nM]	FP - IC_50_ [nM]	FP - IC_50_ [nM]	FP - IC_50_ [nM]	FP - IC_50_ [nM]	DSF - ΔT_m,max_ [ºC]	DSF - ΔT_m,max_ [ºC]
	Competitive 22-NBD-chol	Competitive 25-NBD-chol	Competitive 25-NBD-chol	Competitive 22-NBD-chol	Competitive 22-NBD-chol	Competitive 22-NBD-chol	Competitive 22-NBD-chol		
4β-HC	1,684	>10,000	>10,000	>10,000	>10,000	>10,000	>10,000		
7a-HC	361	9,234	>10,000	>10,000	>10,000	>10,000	>10,000		
7b-HC	838	>10,000	>10,000	>10,000	>10,000	>10,000	>10,000		
7-KC	189	1,578	1,154	306	2,482	4,418	>10,000		
20 (*S*)-HC	41	1,190	1,308	1731	721	2,839	4,472		
22 (*R*)-HC	>10,000	8,022	2,853	9,187	1,582	>10,000	>10,000		
24 (*S*)-HC	213	3,332	2,541	>10,000	7,619	>10,000	6,205		
25-HC	13	882	1,159	622	1895	>10,000	997	2.2	1.3
27-HC	124	1,062	2,736	>10,000	3,369	>10,000	4,207	3.5	1.2
CT	6,192	>10,000	>10,000	>10,000	>10,000	>10,000	>10,000		
CA	>10,000	>10,000	>10,000	>10,000	>10,000	>10,000	>10,000		5.0
CDCA	>10,000	>10,000	>10,000	>10,000	>10,000	>10,000	>10,000	0.5	3.3
DCA	>10,000	>10,000	>10,000	>10,000	>10,000	>10,000	>10,000		7.5
HDCA	>10,000	>10,000	>10,000	>10,000	>10,000	>10,000	>10,000		3.7
AD	> 10,000	> 10,000	>10,000	>10,000	2,704	>10,000	>10,000		
DHEA	>10,000	>10,000	> 10,000	672	656	> 10,000	>10,000		
PRG	>10,000	>10,000	>10,000	417	822	>10,000	>10,000		
21-AcP	>10,000	>10,000	>10,000	1932	801	>10,000	>10,000	2.5	1.3
3-AcP	999	7,258	>10,000	>10,000	3,941	>10,000	6,060		
DHEA-Ac	5,529	>10,000	9,315	>10,000	>10,000	>10,000	>10,000		
U18666A	>10,000	>10,000	7,817	2,319	931	3,537	>10,000		
T	>10,000	>10,000	>10,000	>10,000	>10,000	>0,000	>10,000	1.6	1.1
Me-DHT	> 10,000	>10,000	>10,000	>10,000	>10,000	>10,000	>10,000		
PG	>10,000	>10,000	>10,000	>10,000	1971	>10,000	>10,000	3.3	3.5
TP	>10,000	4,415	7,160	>10,000	762	>10,000	6,527	4.3	3.0
6-DHT-Ac	>10,000	9,996	>10,000	>10,000	1,486	>10,000	>10,000	4.5	3.7

FP = fluorescence polarization; DSF = differential scanning fluorimetry. All data is the mean of three biologically independent experiments. All ligands show an IC_50_ > 10,000 nM for STARD4 as assessed by FI and were therefore removed from the table. The definition of all compound abbreviations can be found in Supplemental Table S1.

In previous studies, contradictory results regarding the binding preference of STARD5 were suggested. While some report the binding of cholesterol and some oxysterols, our results show no binding of cholesterol to STARD5 in aqueous solution. Alternatively, other reports described its binding to bile acids (36, 37). To obtain further insights into STARD5’s binding preferences, we employed our STP screening panel. The results suggest exclusive binding of cholic acid derivatives to STARD5, with DCA displaying the highest ΔT_m,max_ (7.5°C) (Table 4). Furthermore, no other STP in our assay panel bound cholic acid derivatives, suggesting a specific function of STARD5 in bile acid distribution. Docking studies revealed a distinct interaction pattern between STARD5 (pdb: 2r55) (20) and cholic acid. While the 3-OH-group is predicted to interact with V68 and the carboxylic acid with T103, the OH-groups on the B- and C-ring interact with R76. The nonplanar shape of CA due to its *cis* A/B ring fusion seems to orient the molecule in a favorable distance to R76 and the backbone of V68. This might result in an unfavorable orientation for ligands with more planar conformations, providing a plausible explanation for weak (25-HC and 27-HC) and no binding of other oxysterols to STARD5 (Supplemental Fig. S10).

**Fig. 5:**
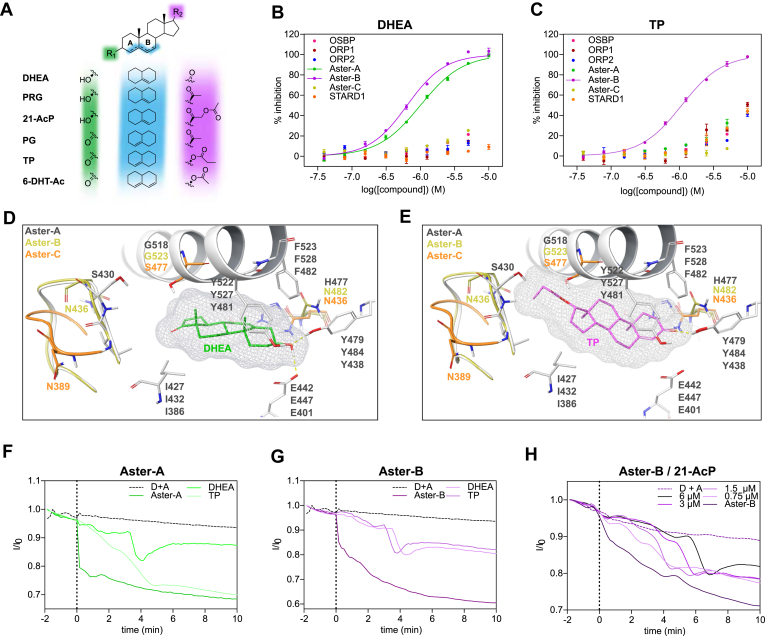
Biophysical screening platform reveals binding of steroid hormone precursors to Aster-B. (A) Chemical structure of the Aster-A and Aster-B binding endogenous and synthetic steroid hormones. (B) Dose-dependent inhibition of STPs binding to NBD-chol by DHEA as assessed by FP. One representative experiment is shown from three independent experiments (n = 3). (C) Dose-dependent inhibition of STPs binding to NBD-chol by TP as assessed by FP. One representative experiment is shown from three independent experiments (n = 3). (D) Representation of the most conserved binding pose of DHEA docked into the crystal structures and homology models of the Asters. (E) Representation of the most conserved binding pose of TP docked into the crystal structures and homology models of the Asters. (F) Inhibition of sterol transport mediated by Aster-A (125 nM, left) by DHEA and TP. One representative experiment is shown from two independent experiments (n = 2), D = donor liposomes, A = acceptor liposomes. (G) Inhibition of sterol tRansport mediated by Aster-B (125 nM, left) by DHEA and TP. One representative experiment is shown from two independent experiments (n = 2), D = donor liposomes, A = acceptor liposomes. (H) Inhibition of sterol transport mediated by Aster-B (125 nM, left) by different concentrations of 21-AcP. One representative experiment is shown from two independent experiments (n = 2). A, acceptor liposomes; D, donor liposomes.

Steroidogenesis is a highly complex multienzyme process converting cholesterol into biologically active steroid hormones, which is largely confined to the adrenal cortex, testicular Leydig cells, ovarian granulosa, and theca cells (38). Some STPs including Aster-B as well as STARD1/3/4/5 are highly expressed in steroidogenic tissues (39). To gain further insight into the binding capabilities of STPs to endogenous and synthetic steroid hormones and their precursors, we evaluated a set of 26 ligands (Table 4). STARD1 reportedly transfers cholesterol from the outer mitochondrial membrane to the inner mitochondrial membrane, initiating its conversion to pregnenolone, and thereby mediating an acute steroidogenic response (40). However, none of the tested ligands bind STARD1 with an IC_50_ < 5 μM suggesting that it is not involved in the direct transfer of those steroid hormones and their precursors. It is thus also unlikely that STARD1 participates in a negative feedback loop where steroidogenic products inhibit their own synthesis by limiting cholesterol availability. In previous studies, 21-Acetoxypregnenolone (21-AcP) was shown to inhibit steroid synthesis in murine MA-10 Leydig tumor cells, with STARD1 as the proposed target (41). Surprisingly, our data suggests that Aster B, and possibly Aster-A, are the primary targets for 21-AcP. Our STP screening panel revealed Aster-A and Aster-B’s equipotent binding of dehydroepiandrosterone (DHEA) (Fig. 5B), pregnenolone (PRG), and 21-AcP all harboring a 3-OH-group as well as a 5,6-alkene in the B-ring (Fig. 5A). Acetylation of the 3-OH-group thereby seems to be less tolerated although alkylation is tolerated as exemplified by the pan-Aster inhibitor U18666A (42). Interestingly, there is stereospecificity in the binding to the Asters as the enantiomer of U18666A was inactive against all STPs in the panel (43). Progesterone (PG), testosterone propionate (TP) (Fig. 5C), and 6-dehydrotestosterone acetate strongly bind Aster-B only, harboring a 3,4,5-*ɑ,β*-unsaturated ketone in the A-ring (Fig. 5A).

When comparing the sterol-binding pockets of Aster-A, -B, and -C, only a few differences can be observed. To rationalize this selectivity profile, we modeled DHEA as well as TP into the Aster-binding pockets (Aster-A pdb: 6gqf; Aster-B homology model based on 6gqf; Aster-C pdb: 7azn; Fig. 5D, E) (13, 42). Whereas DHEA forms key interactions with E442/447 and Y479/484 and perfectly embeds into the Aster-A and -B–binding pocket (Fig. 5C), TP forms interactions with Y484 only and the sterol core is significantly rotated, inducing a possible steric clash with H477 in Aster-A. Furthermore, Aster-C contains a serine (S477) in the middle of the binding pocket instead of a more spacious glycine in Aster-A (G518) and Aster-B (G523), suggesting a steric clash with the sterol core as a plausible explanation for its weak to no binding of the tested ligands. Next, we investigated the effect of DHEA and TP on the Aster-A- and Aster-B-mediated transport of 23-BODIPY-cholesterol (TF-chol) between artificial liposomes, by monitoring changes in FRET FI signal with rhodamine 1,2-dihexadecanoyl-sn-glycero-3-phospho-ethanolamine (Fig. 5, F–H). While Aster-A-mediated TF-chol transport is only inhibited by DHEA (Fig. 5f), Aster-B-mediated transport is inhibited by DHEA and TP (Fig. 5G) confirming the differential binding preferences of those ligands. Interestingly, despite using a large excess of ligand, no full inhibition of transport could be achieved. Furthermore, we also observed the dose-dependent inhibition of Aster-B-mediated TF-chol transfer by 21-AcP (Fig. 5H). Interestingly, for all investigations into the inhibition of Aster-mediated sterol transfer, there is a dose-dependent lag-time from the point at which protein-ligand addition occurs and when the rate of transfer increases steeply. As the competing steroid is present in excess, the binding to the STP is initially saturated, and no transport of TF-chol is observed. However, due to the possible integration of the steroid into the membrane, the transfer of TF-chol proceeds quickly once the competing steroid has been integrated. As such, the data may reflect two processes: a) ligand-protein inhibition in solvent and b) ligand extraction from the membrane. This is further supported by reports showing that sterols and steroidal compounds including PRG, PG, and DHEA can be embedded into lipid bilayers (44, 45, 46). To test this theory, two systems were compared: 1) as outlined above, the protein and ligand were pre-incubated for two minutes and then added to the liposome mixture; and 2) the ligand was preincubated with the liposomes for two minutes, followed by the addition of protein. In the second approach, unlike the first, the sterol transfer is initiated nearly immediately, however, similarly to the first, the maximal inhibition never approaches 100% (Supplemental Fig. S11). This test supports the idea that ligands both act as inhibitors of endogenous sterol transfer, as well as substrates directly from liposomes.

## Discussion

Small molecules and natural products are widely used as powerful tools to explore the specific functions of proteins in the human cell. However, using poorly characterized and nonselective probes can lead to misleading and inconclusive results. In the last decades, a growing set of fluorescent and nonfluorescent sterol derivatives for studying sterol transport has become available. Those probes were extensively studied regarding their localization, interaction, and trafficking in cells (47, 48, 49). However, the majority of those tool compounds have not been profiled with regards to their selectivity towards sterol-binding proteins, leading to inaccurate or incomplete interpretation of biological results. Therefore, we set out to exploit our STP screening panel to characterize different fluorescent sterols regarding their binding and selectivity profiles towards ten STPs. We found that especially the labeling position as well as the linker properties are critical for the observed binding preferences. For example, the widely used 22-NBD-chol binds the majority of STPs in our screening panel, whereas 25-NBD-chol selectively binds to the ORP family proteins. In contrast to that, TF-TMR-chol harboring an amide in the linker has a 6-fold lower k_d_ towards OSBP in comparison to ORP1 and a 30-fold lower k_d_ in comparison to ORP2 and is inactive on the other STPs. We therefore wish to highlight the importance of carefully choosing the probe and the applied concentration for a specific research question. For this, we envisage that the data provided herein will be a valuable resource to guide and facilitate the interpretation of biological results within the field. Furthermore, we identified the inherent fluorescent sterols 25-HCTL and 27-HCTL as potent inhibitors of STARD3, STARD4, and STARD5 and were able to exploit 27-HCTL for the development of a FI-based competitive assay, which will facilitate the high-throughput screening for potent and selective STARD4 inhibitors in the future. However, with insufficient assay windows and Z′-factors 25- and 27-HCTL are not suitable for competitive fluorescence-based assays for STARD3 and STARD5, suggesting that the observed increase in fluorescence upon binding is highly dependent on the specific interaction between compound and protein. Here, DSF serves as an alternative method to detect and evaluate binding to STARD3 and STARD5.

Within the last decades, various studies reported binding data of cholesterol as well as different steroids to STPs; however, information is distributed across a diverse range of sources, thus making it difficult and tedious to find, evaluate, and compare results. By developing a comprehensive set of biophysical tools, we were able to test a set of 42 steroid-based natural products and derivatives including cholesterol. Our results are complemented by a thorough docking-based structural analysis, identifying crucial amino acids in the STP-binding pockets as molecular basis for their individual ligand selectivity. In general, it should be noted that due to the properties of the specific STPs and their differential binding affinities to the fluorescent probes, different protein concentrations had to be used in the different assay systems, influencing the individual assay limits and sensitivities. Furthermore, the poor solubility of hydrophobic molecules like cholesterol makes the determination of accurate binding constants in solution challenging. In the past, several groups managed to avoid those challenges by using a bead-based binding assay with radiolabeled ligands as tracer molecules (9, 13, 50). However, due to the lack of radiolabeled ligands as well as high costs, those assays are not always suitable for mid- or high-throughput screening, while our biophysical screening platform serves as a valuable tool to test and evaluate STPs binding selectivity in a high-throughput manner. All in all, our results prove that STPs are characterized by significant structural differences resulting in differential binding affinities to cholesterol in aqueous solution. Several studies report additional STP-specific mechanisms regulating cholesterol transport in cellular environment. ORP1, for example, is reported to bind PIPs that allosterically enhance ORP1-mediated transport of major lipid species such as cholesterol, while ORP2 co-exchanges cholesterol with PI(4,5)P_2_ in the plasma membrane and cholesterol transport could be facilitated by PIP binding (21, 51). Furthermore, STARD4 favors interactions with anionic membranes through a surface-exposed basic patch directing its membrane specificity (52). These results illustrate the importance of employing multiple techniques to investigate differences in ligand-binding affinity and specificity of STPs in distinct environments (in vitro, aqueous, and liposome-based environment, as well as *in cellulo*). By screening 41 steroid-based natural products, we confirm that small changes on the steroid core, like a differential hydroxylation pattern, can result in varying binding affinities towards the STPs even within the same protein family. For example, OSBP tolerates hydroxylation directly at the steroid core as well as side-chain hydroxylation, while the other STPs mainly tolerate side-chain hydroxylation. Furthermore, our results confirm that in aqueous solution, STARD5 binds bile acids with higher affinity than cholesterol, which could be explained by the presence of an Arg in the center of the pocket forming more favorable key interactions. Additionally, we were able to provide a plausible link between the inhibition of steroid synthesis in murine MA-10 Leydig tumor cells by identifying Aster-A and Aster-B as primary targets for 21-AcP, which was previously suggested to target STARD1. In particular, Aster-B is highly expressed in steroidogenic tissues. By identifying binding of DHEA, PRG and PG as well as their synthetic analogs 21-AcP, 6-dehydrotestosterone acetate, and TP to Aster-B, our results suggest that Aster-B might be directly involved in steroidogenesis. This is supported by recent reports which suggest that Aster-B-mediated cholesterol transport from the plasma membrane to the ER and from the ER to mitochondria directly regulates estradiol production (12, 53).

Importantly, our work complements that on the well-studied Niemann-Pick C type 2 (NPC2) protein involved in lysosomal lipid transport, which is reported to bind several sterol-based ligands. A variety of studies clearly confirm NPC2 as a cholesterol binding/transport protein (54, 55). Furthermore, the fluorescent ligands DHE and cholestatrienol are reported to bind NPC2 with high affinity. Studies also show that like most other STPs, sidechain hydroxylations are preferred over modifications at the sterol-core (56). Interestingly, cholesterol sulfate was shown to bind NPC2 with higher affinity than cholesterol, which could be explained by its higher solubility in aqueous solution. Notably, despite NPC2s potent binding of a variety of sterols, no binding of steroid hormones and their precursors as well as bile acids could be observed (54).

In summary, we established a comprehensive set of biophysical assays complemented by robust docking workflows for the evaluation of ligand selectivity towards sterol transport proteins. We utilized those tools to obtain unique insights into the selectivity profiles of cholesterol as well as 41 steroid-based natural products and the underlying molecular basis. In the future, this study will serve as a resource to guide the selection of suitable fluorescent probes for specific research questions and will guide and facilitate the interpretation of cell biological results. Furthermore, the insights into the STP-binding pockets will guide the development of potent and selective probes for the investigation of STP-specific functions in health and disease.

## Data Availability

The data that support the findings of the study are available from the corresponding author upon reasonable request.

## Supplemental data

This article contains supplemental data (24, 25, 32, 43).

## Conflicts of interests

The authors declare that they have no conflicts of interest with the contents of this article.
